# Sex-Specific Microglial Responses to Glucocerebrosidase Inhibition: Relevance to GBA1-Linked Parkinson’s Disease

**DOI:** 10.3390/cells12030343

**Published:** 2023-01-17

**Authors:** Electra Brunialti, Alessandro Villa, Marco Toffoli, Sara Lucas Del Pozo, Nicoletta Rizzi, Clara Meda, Adriana Maggi, Anthony H. V. Schapira, Paolo Ciana

**Affiliations:** 1 Department of Health Sciences, University of Milan, 20146 Milan, Italy; 2 Department of Clinical and Movement Neurosciences, Queen Square Institute of Neurology, University College London, Royal Free Campus, London NW3 2PF, UK; 3 Aligning Science Across Parkinson’s (ASAP) Collaborative Research Network, Chevy Chase, MD 20815, USA; 4 Animal Care Unit, University of Milan, 20146 Milan, Italy; 5 Department of Pharmaceutical Sciences, University of Milan, 20146 Milan, Italy

**Keywords:** microglia, shape descriptors, glucocerebrosidase (GCase), Parkinson’s Disease (PD), sex-difference

## Abstract

Microglia are heterogenous cells characterized by distinct populations each contributing to specific biological processes in the nervous system, including neuroprotection. To elucidate the impact of sex-specific microglia heterogenicity to the susceptibility of neuronal stress, we video-recorded with time-lapse microscopy the changes in shape and motility occurring in primary cells derived from mice of both sexes in response to pro-inflammatory or neurotoxic stimulations. With this morpho-functional analysis, we documented distinct microglia subpopulations eliciting sex-specific responses to stimulation: male microglia tended to have a more pro-inflammatory phenotype, while female microglia showed increased sensitivity to conduritol-B-epoxide (CBE), a small molecule inhibitor of glucocerebrosidase, the enzyme encoded by the GBA1 gene, mutations of which are the major risk factor for Parkinson’s Disease (PD). Interestingly, glucocerebrosidase inhibition particularly impaired the ability of female microglia to enhance the Nrf2-dependent detoxification pathway in neurons, attenuating the sex differences observed in this neuroprotective function. This finding is consistent with the clinical impact of GBA1 mutations, in which the 1.5–2-fold reduced risk of developing idiopathic PD observed in female individuals is lost in the GBA1 carrier population, thus suggesting a sex-specific role for microglia in the etiopathogenesis of PD-GBA1.

## 1. Introduction

Microglia are resident myeloid cells playing an essential role in the development and homeostasis of the brain, starting from embryonic development and throughout adult life. The physiological function of microglia includes the well-known innate immune response to pathogenic insults [1], the sculpting of neuronal termination by pruning synapses [2], the engulfment of cellular bodies and debris [3], and the synthesis of communication molecules, growth factors, and neurotransmitter precursors [4], which finally result in a strong influence on synaptic transmission [5]. The fine tuning of these basic biological processes ensures homeostasis and maintains brain tropism, while the presence of dysregulated microglia function is considered a hallmark of neurodegeneration [6]. The full involvement of microglia in the neurodegenerative processes is still the subject of investigation [7], but chronic inflammatory activation may result in neuronal damage [6], and abnormal activation of microglia could contribute to the spread of alpha-synuclein and beta-amyloid plaques in the brain of PD and Alzheimer’s disease (AD) patients [8,9]. Microglia can exert different functions in the brain by virtue of their marked plasticity, which allows these cells to acquire a wide range of morphological phenotypes, each characterized by different functional properties; these phenotypes can be triggered by specific stimuli, such as pro- and anti-inflammatory cytokines [10,11], but are also influenced by the surrounding microenvironment, where the activity of microglia is directed by endocrine [12] and paracrine signals [13]. For these multi-functional abilities, in the different brain areas, heterogenous microglial subpopulations co-exist at the same time [14,15]. Interestingly, a further level of microglia heterogenicity is due to genetic determinants, including sex-dependent factors which influence both microglia distribution in the central nervous system (CNS) [16,17] and some cell-specific morpho-functional properties [18], which microglia retain even when transplanted into the brain of the opposite sex [19]. The differential response to stimulation [18,20] of female versus male microglia has been hypothesized to contribute to the sex-dependent bias observed in the prevalence of certain neurological diseases [12,19,21], in particular AD and PD for which sex is considered an unmodifiable risk factor [22,23]. Female sex is a risk factor for AD [23] and multiple sclerosis [24], while male sex is a risk factor for motor neuron disorders [25] and PD [22]. In this context, another clinically relevant (genetic) risk factor for PD is the presence of specific mutations in the GBA1 gene, which have been detected in up to 5–25% of patients [26,27]. This gene encodes for a lysosomal hydrolase, namely, the glucocerebrosidase (GCase): biallelic mutations in GBA1 cause Gaucher Disease (GD) [28], while heterozygotic carriers do not develop GD but retain the increased risk to develop PD [29]. Although most studies previously focused on the functional effects of GBA1 mutations in neurons, our recent investigations revealed that GCase inhibition in microglia is sufficient to impair the physiological ability of microglial to protect neurons against oxidative stress and neurotoxic stimuli [30]: this acquired microglia phenotype may contribute to the increased risk of neurodegeneration observed in GBA1 carriers.

To investigate the microglial phenotype due to GCase inhibition, in the current study, we developed and applied a non-invasive imaging approach to primary cultures generated by murine models of both sexes. This original methodology allowed us to record in real time the changes in cell morphology induced by specific pharmacological stimuli, with the aim of associating the dynamic variation in cell shape and motility to the biochemical effects induced by the treatments. With this analysis, we found that the effects of GCase inhibition in microglia are sex-dependent, thus showing a greater loss of the neuroprotective ability of female microglia compared with male microglia.

## 2. Materials and Methods

### 2.1. Cell Cultures 

Primary neurons were derived from the cerebral cortex of p0–p1 mice following standard operational procedure using the neural tissue dissociation kit—postnatal neurons (Cat. 130-094-802, Miltenyi Biotec, Bergisch Gladbach, North Rhine-Westphalia, Germany), as previously described [30]. In brief, the brain cortices from 6 mice of both sexes were pooled as a single experimental group and subjected to enzymatic and mechanical dissociation, then 150,000 primary neuronal cells were seeded for each well of a poly-L-ornithine-coated 24-well plate, replacing half of the medium volume every 2 or 3 days. At day 10, 37,500 primary microglia cells isolated from the whole brain of adult male or female mice (age 3–6 months) were seeded on a neuron layer [19]; briefly, the brains from two mice were pooled and subjected to enzymatic and mechanical dissociation and microglia were purified using a magnetic column and anti-CD11b-coated microbeads (Cat. 130-093-634, Miltenyi Biotec). Neuronal and microglial cultures were grown in Neurobasal A medium (Cat. 10888-022, LifeTechnologies, Carlsbad, CA, USA) containing 1% streptomycin–penicillin, 1% GlutaMAX, 2% B-27 Supplement (Cat. 17504-044; Gibco, Thermo Fisher Scientific, Waltham, MA, USA), and 10 mM HEPES (Cat. H0887, Merk, Darmstadt, Hesse, Germany), in a humidified 5% CO_2_-95% air atmosphere at 37 °C.

### 2.2. Cell Treatments

For lipopolysaccharide (LPS) experiments, cultures were treated with a final concentration of 10 µg/mL LPS O111:B4 (Cat. L2630, Merk) for 6 h or vehicle (water); for the CBE experiments, cultures were treated with a final concentration of 200 µM CBE (Cat. 234599, Merk) or vehicle (water) for 48 h and then subjected to time-lapse microscopy.

### 2.3. Fluorescent Image Acquisition and Processing

Time-lapse sessions were performed on live microglia for 20 random fields per condition using an Axiovert 200M microscope with AxioVision Imaging System (version 4.9, RRID:SCR_002677, https://www.micro-shop.zeiss.com/it/ch/system/software+axiovision-axiovision+programma-axiovision+software/10221 accessed on 22 March 2022, Carl Zeiss Ltd., Cambridge, UK) at ×20 magnification; the recording was performed for 2 h taking a picture every 5 min. An algorithm was generated to segment and analyze GFP-positive cells (namely, microglia) using Fiji software (version 2.0.0, RRID:SCR_002285, http://fiji.sc accessed on 22 March 2022, ImageJ, NIH, Nature Methods, 2012). The background was subtracted and set constant across the experimental groups; the class of pixels with a value over the defined threshold (foreground) that corresponds to green fluorescent objects were subject to the despeckle and smoothing function. Then, the objects with an area greater or equal to 130 μm^2^ were subjected to the “analyze particle” function to calculate the “Area”, “Center of mass”, “Shape descriptors”, and “Feret’s diameter” for each object in each frame. The area was converted from pixels to the surface in μm^2^; the coordinates of the center of mass of each object were used to calculate the distance covered by the cell during the time lapses. Among the “Shape descriptors”, we operated with the solidity, a value that corresponds to the area/convex area of the object; between the “Feret’s diameter” values we used the “Feret Angle” to calculate the number of rotations of each object during the recording. A math operation was used to perform the clustering analysis: in brief, for each parameter obtained from the analysis, the values of the vehicle and treated cells were used to identify the median parameter for the experiment, this median was used as a threshold to cluster the cells into two groups (over or under the median); the combination of two parameters was used to generate four different clusters.

### 2.4. Animals and Treatments

The animals were fed ad libitum and housed in individually ventilated plastic cages within a temperature range of 22–25 °C under a relative humidity of 50% ± 10% and an automatic cycle of 12 h light/dark. C57BL/6 and CX3CR1^+/GFP^ mice were supplied by Charles River (Charles River LaboratoriesWilmington, MA, USA, RRID:MGI:2159769 and MGI:J:84544) and ARE-*luc2* mice (MGI:7388153) were generated in our laboratory [31]. For pharmacological treatments, mice (15–30 weeks old) were administered 100 mg/kg/day CBE or vehicle (PBS) via i.p. injection for 3 days before the purification of microglia.

### 2.5. Luciferase Enzymatic Assay

Luciferase assays were performed as illustrated previously [32]. In brief, microglia-neuron cultures were lysed with luciferase cell culture lysis reagent (Cat. E1531, Promega, Madison, WI, USA), and the protein concentration was determined with a Bradford assay [33]. The luciferase activity assay was carried out in luciferase assay buffer by measuring luminescence emission with a luminometer (Veritas, Turner, Promega) for 10 s to obtain the relative luminescence units (RLU).

### 2.6. Clinical Data

Clinical data were obtained from the Accelerating Medicines Partnership Parkinson’s Disease (AMP-PD) knowledge portal, downloaded on the 28 May 2020 (release 15 October 2019). Correct GBA sequencing was obtained using Gauchian (Version 1.0.2, https://github.com/Illumina/Gauchian accessed on 26 September 2022), a software described in a previous publication [34]. Participants with the following tags were included: “PD”, “Genetic Registry PD”, “Genetic Cohort PD”, “Genetic Registry Unaffected”, “Genetic Cohort Unaffected”, and “Healthy Control”. Participants marked as “Prodromal” and “SWEDD” (scans without evidence for dopaminergic deficit) were excluded from the analysis.

### 2.7. Statistical Analysis

For the cellular experiment, statistical analyses were performed employing Prism 7 (version 7.00, RRID:SCR_002798, http://www.graphpad.com accessed on 26 September 2022, GraphPad Software Inc., San Diego, CA, USA) and multiple *t*-test versus vehicle were used to determine if there were significant differences in means and a *p*-value lower than 0.05 was considered to indicate statistical significance. For the clinical data, statistical analysis was carried out using R (version 4.2.1, RRID:SCR_001905, http://www.r-project.org accessed on 26 September 2022, Ugeskr Laeger, 2008). Pearson’s Chi-squared test was used to compare sex differences between carriers and non-carriers of GBA variants.

## 3. Results

### 3.1. Image-Based Microglia Analysis Allows Detecting Functional Clusters

**Figure 1:**
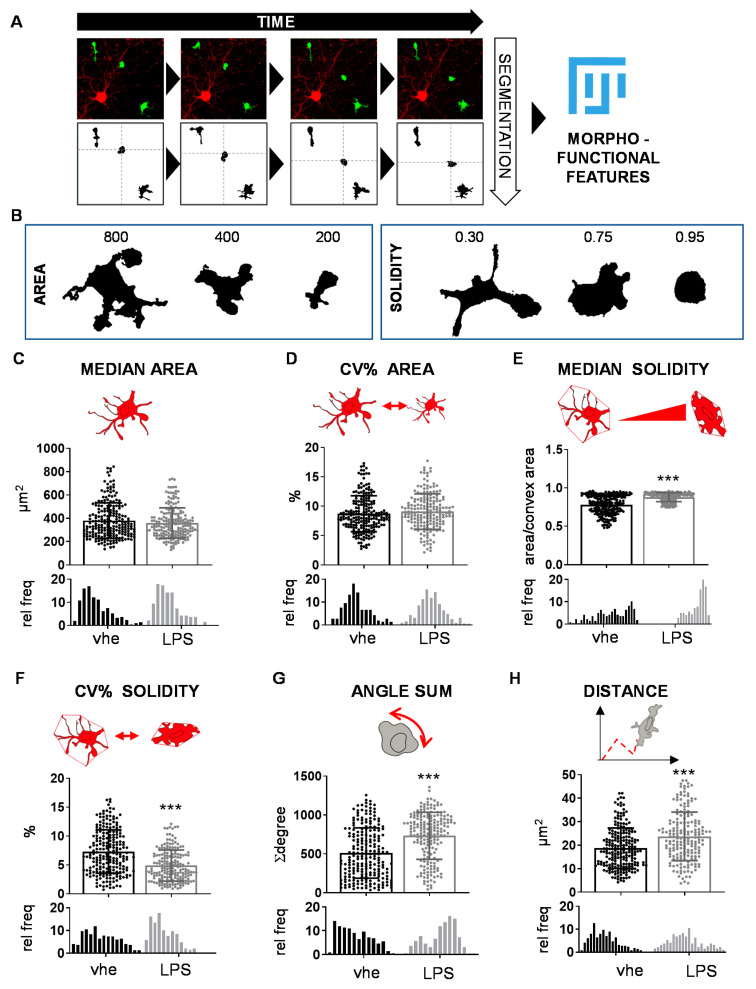
Unbiased morpho-metric method to detect morpho-functional changes. (**A**) Schematic representation of the image-based method for the morpho-functional clustering: GFP-marked microglia dynamics were recorded by time-lapse microscopy, and the acquired images were segmented and processed with Fiji software to obtain shape and dynamic descriptors for each microglial cell during the time lapse period. (**B**) Representative images of shape descriptors (area and solidity) used for the analysis. (**C**–**H**) Quantitative single-parameter analysis of lipopolysaccharide (LPS)-treated male microglia; the analysis includes (**C**) median area, (**D**) coefficient of variation (CV%) of the area, (**E**) median solidity, (**F**) CV% of solidity, (**G**) rotation, and (**H**) distance covered. The values are presented as mean ± SD (top) and as frequency distribution (bottom) of *n* = 3 independent experiments. The drawings are a schematic representation of the parameter reported in the graph. *** *p* < 0.001 calculated by *t*-test versus vehicle.

To investigate the changes in microglia morphology occurring as a consequence of specific stimuli, we generated an unbiased imaging approach allowing for the dynamic quantification of shape and movement variations of single cells over a fixed period of time. To mimic the physiological microglial environment, we seeded primary adult microglial cells obtained from CX3CR1^+/GFP^ mice, constitutively expressing GFP [35], on a layer of neuron-enriched primary culture of cortical cells from syngeneic wild-type mice (Figure S1) known to structurally support microglia [19]. The bias of the neuronal layer was avoided by using a mix of female and male neurons. Time-lapse microscopy allowed the recording of morphology and movements of GFP-positive microglia over 2 h; the recorded movies were processed with the ImageJ software [36] to obtain morphological and kinetic descriptors for each cell in the acquired field of view (Figure 1A, Video S1). Briefly, the background was subtracted from the acquired images, which were, in turn, binarized using a defined threshold that enabled cluster regions of pixels based on the similarity threshold to distinguish cell shapes and generate an object for each cell. Then, the binarized images were processed to remove noise by using the smooth and despeckle functions of the software to produce sharp objects.

A threshold of 130 µm^2^ for the surface size was selected to sort the shapes of cells (microglia) from those originating from cellular debris. The selected shapes were processed to measure two static morphology descriptors: the cell area in square micrometers and the solidity (Figure 1B) [37]. The latter is defined as the ratio of the area divided by the area of the smallest convex set polygon that contains the cell [36], thus resulting in a higher solidity for ameboid rather than for ramified shapes, in a range from 0 to 1 values (Figure 1B). For each cell, measurements were taken in every frame of the time-lapse acquisition; median values of these measurements described the predominant morphology during recording and were used to generate the graphs (Figure 1C,E). Coefficients of variation (CV%) for area and solidity were calculated to obtain numeric descriptors of the dynamic changes occurring during the 2 h measurements (Figure 1D,F). The CV% of the cell area due to the size variation was taken as a surrogate marker of cell contractility, while the CV% of the solidity was considered as a measure of the morphological modifications in terms of complexity.

Since microglia are cells able to sense the environment and migrate in response to specific stimuli [38], the distance traveled by microglia during the recording time was also considered as a parameter inherently linked to their activity: distance was calculated by tracing the shift in the center of mass of each cell occurring frame by frame, in terms of coordinates (x, y). To define the total covered distance, all shifts were summed and converted into µm values (Figure 1H). Finally, we measured the number of rotations performed by each cell, which is another descriptor representing microglial dynamics. In order to calculate this parameter, the ellipse in which the cell can be inscribed was identified and used to calculate the angular displacements frame-by-frame, which were, in turn, added up to obtain the total rotation of the cells during the recording, expressed as angular degree values (Figure 1G).

To validate the method, we analyzed the descriptor changes associated with a well-characterized microglia polarization, namely, the one caused by the potent endotoxin lipopolysaccharide (LPS) [39], a strong inducer of a pro-inflammatory microglial phenotype [40]. Male-derived microglial cells were cultivated on the layer of primary neuron-enriched cultures for 24 h and treated with 10 µg/mL LPS. Microglia morphology and motility were analyzed and compared with vehicle-treated cells by processing videos captured from 6 h up to 8 h after the treatment, a time point that is associated with high gain of pro-inflammatory features [41]. The experiment revealed that the selected descriptors were effective in detecting and describing specific features of microglia induced by LPS (Figure 1 and Video S2) [42,43]. In detail, the area of the analyzed cells did not change during the acquisition (Figure 1C,D), but the treatment induced an increase in their solidity of about 13% meaning that when microglia were treated with LPS, their shape became more ameboid (Figure 1F), while cells maintained their complexity across time, since the variation of solidity (CV% solidity) was higher in vehicle-treated and lower in LPS-treated cells (Figure 1G). As expected, the cell kinetics were also affected by LPS, showing an increase of about 43% in the number of rotations (Figure 1G) and of about 27% in the covered distance (Figure 1H) when compared with vehicle-treated cells.

**Figure 2:**
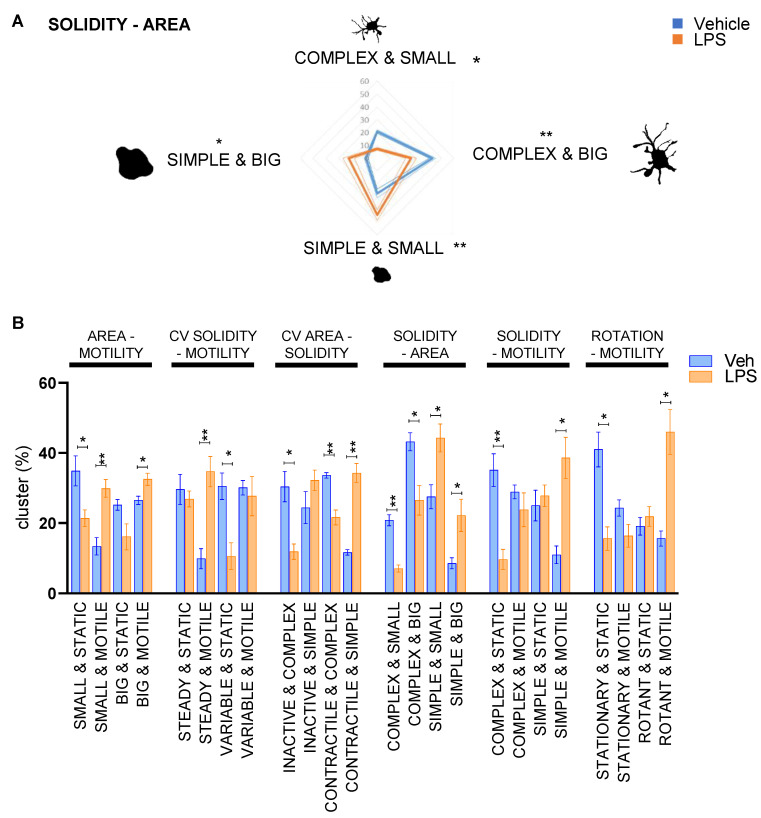
Morpho-functional biparametric analysis of LPS-treated male microglia. (**A**) Biparametric analysis of microglial solidity and area allows to cluster the cells into four categories: “complex & big”, “complex & small”, “simple & small”, and “simple & big”. The percentage of each population is reported as a radar graph relative to three independent experiments. Drawings represent the morphology representative of the category. * *p* < 0.05, and ** *p* < 0.01; calculated by *t*-test versus vehicle. (**B**) Biparametric analysis represented as histogram of the median ± SEM of the subpopulation percentage obtained in 3 different experiments; the two parameters obtained to generate the clusters are reported on the top of the graph. * *p* < 0.05, and ** *p* < 0.01 calculated by *t*-test versus vehicle.

The results showed that the single-parameter analysis was efficiently identifying phenotypic changes induced by a strong stimulus—as potent as LPS is—occurring in the overall microglial population, but did not provide any detail on the presence of microglia subpopulations with different behaviors (Figure 1A–H, Video S2). This is particularly important, since microglia shows a peculiar heterogeneity in physiological conditions suggesting the existence of various subpopulations reacting differently upon stimulation [44,45]; we attempted to discriminate these different microglia subpopulations by combining our cellular descriptors in a cluster analysis. We performed a biparametric analysis to test whether we could distinguish the existence of distinct morpho-functional categories: the medians of morpho-dynamic descriptors were used as a cutoff to assign each cell to a descriptive category representative of a value above or under the media. By using the combination of two parameters, cells were clustered into four different subpopulations. As an example, by analyzing solidity and area (Figure 2A) it was possible to generate four clusters representing microglia subpopulations: Cluster 1 “simple & big”, Cluster 2 “simple & small”, Cluster 3 “complex & big” and Cluster 4 “complex & small”. Cluster 1 is composed of cells that have both area and solidity above the median. In contrast, the cells with area and solidity under the median fall in Cluster 4; cells with a bigger area and low solidity fall in Cluster 3; and small and simple cells are in Cluster 2.

The categories generated for each parameter are reported in Table 1 and Table S1. The application of this approach to the data obtained with the LPS experiment, in keeping with previous reports [19,42,43,46], revealed that after treatment a population of cells characterized by a “complex & small” shape mostly disappeared, while an increase in the subpopulation of “simple & small” cells was observed (Figure 2A) and the subpopulation of “simple & big” cells, which was under-represented in the vehicle-treated samples, became prominent after LPS treatment (Figure 2A). The cluster analysis was applied to identify different morpho-functional subpopulations and was reported in specific histograms (Figure 2B) demonstrating how the different subpopulations were affected by the LPS treatment. The graphs show that LPS treatment increased the subpopulation of “small & motile”, ”big & motile”, “steady & motile”, “contractile & simple”, “simple & small”, “simple & big”, “simple & motile”, and “rotant & motile” (Figure 2B). Interestingly, the method was able to also detect that some categories of cells did not respond to LPS and their subpopulation remained unaffected after the treatment, e.g., “steady & static”, “variable & motile”, “complex & motile”, and “simple & static” (Figure 2B). These results demonstrated that the dynamic morpho-functional analysis allowed to discriminate microglial subpopulations differentially responding to specific stimulations.

### 3.2. Male and Female Microglia Show Different Morpho-Functional Phenotypes

**Figure 3:**
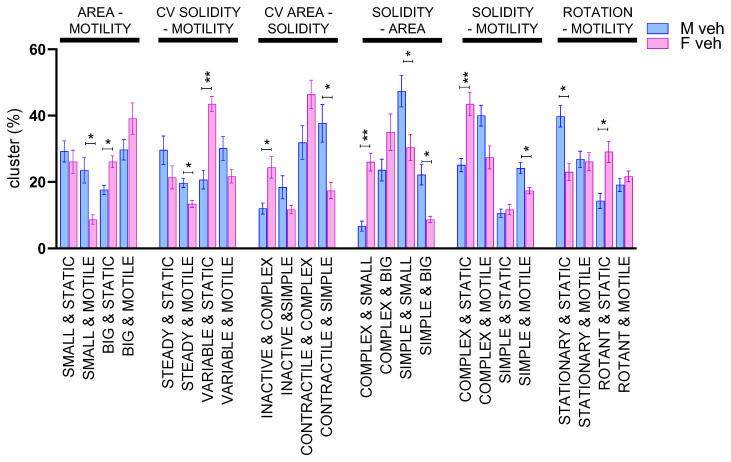
Morpho-functional biparametric analysis of male and female microglia. Biparametric analysis is represented as a histogram of the median ± SEM of the subpopulation percentage obtained in 3 different experiments. The two parameters obtained to generate the clusters are reported on the top of the graph. * *p* < 0.05, ** *p* < 0.01 calculated by *t*-test versus vehicle.

Once validated, the morphometric approach was used to test whether sex differences could be detected in the dynamic behavior of microglia. To this end, primary brain microglia cells from male or female mice were isolated from adult CX3CR1^+/GFP^ and seeded on neuron-enriched primary cortical cells from syngeneic wild-type mice (mixed population of male and female mice). After 24 h of seeding, microglia dynamics were recorded for 2 h to identify possible sex-related differences in unstimulated conditions. Indeed, different subpopulations were present in female and male microglia: when compared with male, female microglia showed cluster subpopulations of “big & static”, “variable & static”, “inactive & complex”, “complex & small”, ”complex & static”, and “rotant & static” (Figure 3). These data suggest that female microglia in physiological conditions are enriched in subpopulations characterized by complex and static cells, possibly interacting with the surrounding microenvironment, with a less pro-inflammatory profile. This is in accordance with previous reports indicating that, in female mice microglia are more dedicated to the maintenance of brain homeostasis, while male microglia are more inclined to perform defensive tasks [19].

### 3.3. Chemical Inhibition of β-Glucocerebrosidase (GCase) Exerts a Differential Effect in Male and Female Microglia

**Figure 4:**
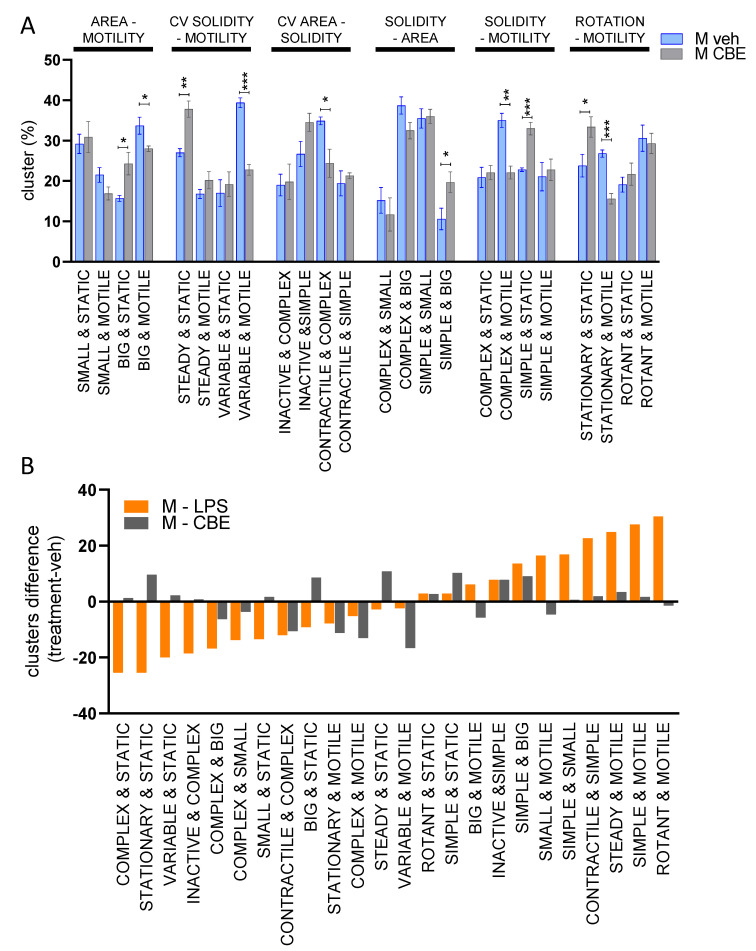
Morpho-functional biparametric analysis of CBE-treated male microglia. (**A**) Biparametric analysis is represented as a histogram of the median ± SEM of the subpopulation percentage obtained in 4 different experiments, the two parameters obtained to generate the clusters are reported on the top of the graph; * *p* < 0.05, ** *p* < 0.01, *** *p* < 0.001 calculated by *t*-test versus vehicle. (**B**) Variation in subpopulation composition for LPS (Figure 2B) and CBE (**A**) treatment versus the vehicle-treated cells.

We previously demonstrated that the pharmacological inhibition of microglial GCase with conduritol-B-epoxide (CBE) interferes with the neuroprotective function of microglia [30]. To characterize the microglia morphology in response to GCase inhibition, we carried out the morpho-functional analysis after treating cocultures with 200 µM CBE: this concentration was selected in order to ensure an almost total (−98% activity) inhibition of GCase activity sufficient to selectively interfere with the microglia neuroprotective functions [30], while with negligible effects on the activity of additional glycosidase targets [47,48]. The dynamic changes in microglial morphology were recorded at early time points (48 to 50 h after treatment), a time window in which neuronal and microglia mortality due to CBE were virtually absent [30]. The morphometric analysis revealed that GCase inhibition of male microglia changed the phenotype of specific subpopulations, increasing cells characterized by a static and less contractile phenotype (Figure 4A). We observed increases in cell populations with “big & static”, “steady & static”, “simple & big “, “simple & static”, and “stationary & static” cells, while a decrease in the subpopulations of cells was observed in “big & motile”, “variable & motile”, “contractile & complex”, “complex & motile”, and “stationary & motile” (Figure 4A). Overall, the phenotype observed was characterized by a large and static morphology (Video S3), inclusive of large and simple shapes associated with a low motility. Since it has been reported that microglia can acquire a pro-inflammatory phenotype after long-term GCase inhibition [49,50], we compared the phenotype triggered by LPS (Figure 2B) with the short-term treatment with CBE (48–50 h). Surprisingly, the morpho-functional analysis of male microglia treated with LPS or CBE (Figure 4B) demonstrated opposite effects by increasing (LPS) or decreasing (CBE) the motility in most subpopulations.

**Figure 5:**
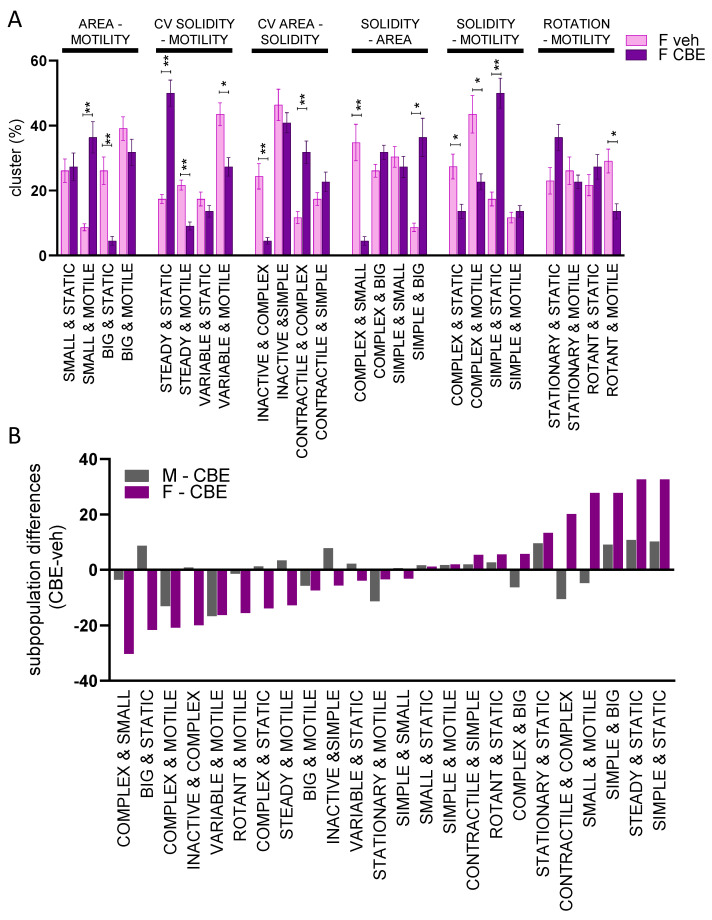
Morpho-functional biparametric analysis of CBE-treated female microglia. (**A**) Biparametric analysis represented as a histogram of the median ± SEM of the subpopulation percentage obtained in 3 different experiments. The two parameters obtained to generate the clusters are reported on the top of the graph. * *p* < 0.05, ** *p* < 0.01 calculated by *t*-test versus vehicle. (**B**) Variation in subpopulation composition for CBE-treated male (Figure 4A) and CBE-treated female (**A**) microglia versus the vehicle-treated cells.

A sex-difference in microglial reactivity has been previously described [19], so we investigated if the CBE treatment differentially affected the microglia phenotype obtained from female or male mice. Thus, we treated cocultures of female microglia with CBE and recorded the effects at the same time points (48 to 50 h after treatment) as the previous experiment. As reported in Figure 5A, GCase inhibition induced a radical shift in female microglia morpho-functionality, leading to an increased representation of subpopulations of “small & motile”, “steady & static”, “contractile & complex”, “simple & big”, and “simple & static” and a decrease in the subpopulations of “big & static”, “steady & motile”, “variable & motile”, “inactive & complex”, “complex & small”, “complex & static”, “complex & motile”, and “rotant & motile” (Figure 5A) cells. Comparison of the results obtained with female (Figure 5A) and male microglia (Figure 4A) after CBE treatment revealed that some subpopulations were differentially enriched, and that CBE treatment induced more marked morpho-functional changes in female microglia (Figure 5B).

**Figure 6:**
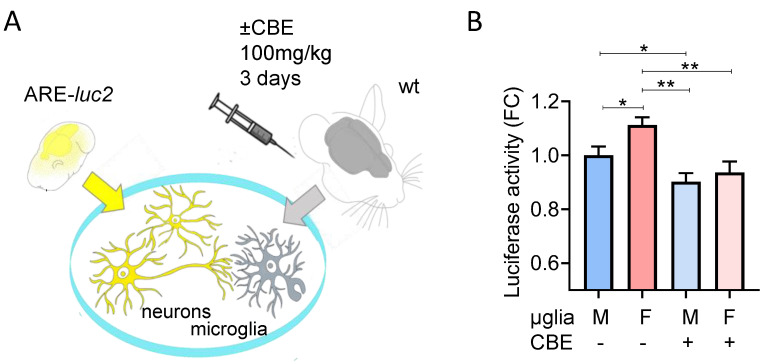
CBE treatment reduces the neuronal Nrf2 response induced by microglia. (**A**) Scheme of the experiments reported in B aimed at testing the effect of primary microglia (µglia) extracted from male or female mice treated with CBE. (**B**) Luciferase activity measured in protein extracts derived from ARE-*luc2* neurons cultured with microglia derived from CBE- or vehicle-treated mice (**A**). Data are presented as mean ± SEM of *n* = 7 independent samples. * *p* < 0.05, ** *p* < 0.01 calculated by unpaired *t*-test vs. the corresponding sample.

Based on these results, we decided to test whether this more pronounced effect of CBE on female microglia also reflected alterations in their neuroprotective functions; indeed, we previously demonstrated that microglia are able to increase neuronal NRF2 transcriptional activity that protect neurons from neurotoxin effects, a mechanism which is impaired by GCase inhibition [30]. We purified microglia from groups of female and male wild-type mice treated with vehicle or 100 mg/kg CBE for 3 days to inhibit microglial GCase [30]; the purified microglia were seeded over a neuronal cell layer derived from ARE-*luc2* mice (Figure 6A), transgenic animals in which a luciferase reporter is expressed under the control of the Nrf2 transcription factor [31,32]. This system allowed us to measure the ability of microglia to increase the neuronal Nrf2 activity simply by measuring the luciferase activity in the coculture. Interestingly, female microglia extracted from vehicle-treated mice revealed a more prominent effect in inducing Nrf2 response when compared with male microglia. The effect of CBE treatment, which is expected to reduce neuronal to microglia Nrf2 response [30], was sufficient to blunt the differences observed between male and female microglia obtained from vehicle-treated mice (Figure 6B), thus suppressing the neuroprotective action exerted by microglia independently from the sex of origin.

Based on these data, which suggest that a reduction in GCase activity decreases the protective microglial response in female mice, we hypothesized that the normal male predominance seen in PD patient populations would be abolished in PD subjects with GBA1 variants.

**Figure 7:**
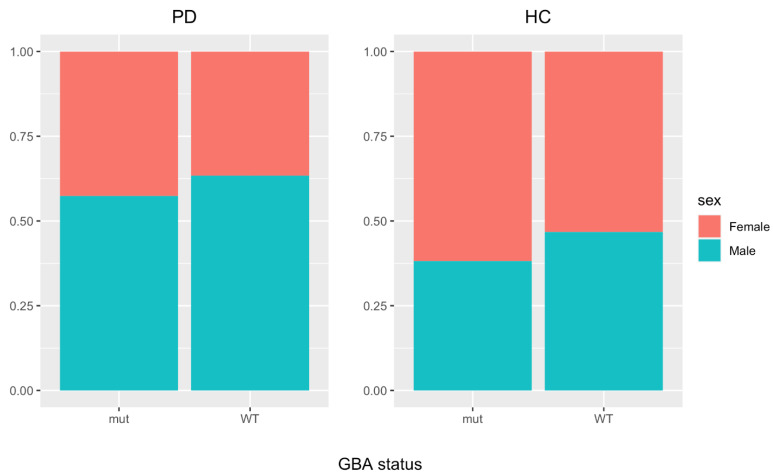
Different sex distribution in idiopathic-PD and GBA-PD patients. The plot summarizes the ratio of females and males in PD and healthy control (HC) populations; mut = mutant.

We analyzed the AMP-PD database that includes a total of 3497 individuals (Figure 7). Of these, 1971 (56.4%) were males and 1526 (43.6%) were females. For idiopathic PD cases alone, there were 1236 males (63.4%) and 715 females (36.6%). In the GBA-PD group, there were 163 males (57.4%) and 121 females (42.6%). Statistical analysis showed that the male predominance in idiopathic PD is lost in GBA1-associated PD, although this just fails significance at *p* = 0.0525.

## 4. Discussion

Microglial cells are characterized by the presence of different subpopulations, which differ in abundance and morphology, and are characterized by distinct genetic programs, protein expression patterns, and the ability to respond to environmental stimuli [14,15]. The distribution of these subpopulations follows a spatiotemporal definite pattern: indeed, specific phenotypes can be detected at different evolutionary stages, but they can also coexist simultaneously in the brain parenchyma of adult animals [44,51]. The morphology of microglia is indicative of their functional status [6,10], thus analysis of microglial shape can anticipate information about the biochemical pathways triggered in these cells by pathophysiological processes [52]. Standard morphological analysis based on immunocytochemistry images provides snapshots of cell shape and offers a static view of the cell population [37] but it does not detect dynamic changes, such as the variation in cell protrusions or changes in migration, features that are certainly key components of microglial biology and allow better deciphering of their behavior [53]. In our study, we added the temporal dimension to standard shape analysis by applying time-lapse fluorescence microscopy to our in vitro model of the multicellular condition of the brain, encompassing a co-culture of GFP-expressing primary microglia and primary cortical cells enriched in neurons. In this context, we applied an unbiased imaging-based analysis for each microglia cell of the investigated population, and for each frame of the recorded movies, we measured standard morphological cues that included cell dimension and complexity, together with novel dynamic descriptors able to describe time-dependent changes in microglia motility, contractility, rotation, and complexity. The method was effective for detecting changes occurring in pro-inflammatory microglia, which have been previously described [42,54] and include changes in the cellular shape towards the amoeboid morphology, and a general increase in the motility (Figure 1 and Figure 2).

The first set of experiments was designed to validate the method and the results were in line with prior knowledge, but at the same time revealed information on the response of primary microglia to pro-inflammatory stimuli by disclosing resilient subpopulations of cells that did not undergo substantial changes after stimulation. These subpopulations show phenotypes that—following the classification generated by our protocol—are defined by the descriptors “complex & motile”, “simple & static”, “stationary & motile”, and “rotant & static”, and display a non-responsive phenotype against LPS stimuli (Figure 2B). Thus, our morpho-functional analysis provided a direct demonstration that adult microglia exist in different subtypes, each characterized by peculiar shapes, and possibly by different gene expression profiles and functions [44,52], and that these subtypes can differently respond to stimulations.

Once validated, we have applied the morpho-functional analysis to evaluate sex-related differences in the composition of microglia subpopulations. In previous studies, biochemical, morphological, and functional data recognized sex-related differences in microglia revealing that male microglia have a constitutive mild pro-inflammatory phenotype, while female microglia are reminiscent of surveilling microglia, that for definition are stationary and ramified cells sensing the environment [19,55,56]. These differences were shown to be genetically determined, independent of hormonal status and of the microenvironment; indeed, they are also maintained when microglia are maintained in culture, when cross-transplanted in a brain of opposite sex, and when microglia were extracted from brains of ovariectomized females, where the hormonal environment was similar to male mice [19]. It is likely that these sex-related differences in microglia are contributing to the differential sex-specific susceptibility and severity of some neurological diseases [12,57,58].

In our morpho-functional analysis, male microglia, when compared with female cells were enriched in subpopulations defined by the descriptors “small & motile”, “simple & motile”, “contractile & simple”, “steady & motile”, “small & simple”, and “simple & big” (Figure 3), which are increased when cultures are treated with LPS (Figure 2B), thus supporting the notion that male microglia show a higher tendency to acquire a pro-inflammatory phenotype than female microglia. In contrast, in female microglia we found a marked presence of subpopulations defined by the descriptors “big & static”, “variable & static”, “inactive & complex”, “complex & small”, and “complex & static” (Figure 3), categories that are decreased after LPS treatment (Figure 2B) supporting the hypothesis that female cells show a less pro-inflamed phenotype, with a profile reminiscent of surveilling microglia [10,19].

Do these differences influence the development and progression of a neurodegenerative process? Previous data from our lab showed that microglia may contribute to brain neuroprotection by inducing the Nrf2 pathway in neurons through direct contact between microglial cells and neurons. This Nrf2 activation is reduced when microglial GCase is pharmacologically inhibited, an effect that renders dopaminergic neurons more sensitive to neurotoxic stimulations [30]. On the basis of these data, we hypothesized that the reduced microglial neuroprotective functions might contribute to the observed increased risk of PD in carriers of GBA1 mutations and prompted us to analyze the microglial morpho-functionality after GCase inhibition. Interestingly, the morpho-functional analysis on static descriptors demonstrated that inhibition of GCase enriched the microglial population with cells characterized by an ameboid-like (“simple & big”) morphology such as those observed with LPS (Figure 4) and typical of the pro-inflammatory activation. Similar peculiar microglial morphologies with bigger soma and less protrusion have been detected in murine and vertebrate GCase-deficient models induced by genetic modification [59,60] and in brain areas (such as substantia nigra) of neuropathic GD patients [61,62], and were often associated with a pro-inflammatory phenotype. However, with our morpho-metric analysis, the use of dynamic descriptors clearly distinguished the effects of LPS and CBE treatments on microglia, showing that male CBE-treated cells were static and less contractile, a phenotype markedly different from the pro-inflammatory phenotype (Figure 2B and Figure 4B), and characteristic of inactive microglia. This is in line with our previous expression data showing that no pro-inflammatory genes were induced by the CBE treatment in immortalized microglia [30]. The more stationary phenotype and the decreased number of protrusions suggest that CBE-treated microglia display a reduced contact surface with the neuron membranes, a condition likely contributing to the decreased Nrf2 expression in neurons [30]. The reduction in Nrf2 levels might increase the risk of neurodegeneration especially in neurons of the substantia nigra that are frequently exposed to oxidative stress due to dopamine metabolism [63]. In the case of GBA1 mutations, the microglial GCase inactivation is constitutive and over time could promote pathways leading to the promotion of neurodegeneration.

Interestingly, female microglia seem to be more affected by GCase impairment; indeed, the morpho-functional phenotype is more divergent from the vehicle when compared with male microglia (Figure 5). The CBE effect on female microglia increases the subpopulations characterized by a less active behavior (less ramified shape and static) to a greater extent compared with male microglial cells. Moreover, female microglia also displayed a divergent response compared with male microglia; indeed, CBE treatment increased the subpopulations defined by the descriptors as “small & motile” and “complex & contractile”. To the best of our knowledge, this is the first description that the inhibition of GCase is able to have a different effect on the morphology and motility of male and female microglia.

Intriguingly, the effect of GCase inhibition is more penetrant in female microglia, reducing the superior ability of female microglia to induce Nrf2 in neurons to the same extent found in male microglia (Figure 6). This dramatic change in female microglia function is likely diminishing the greater neuroprotective ability of female microglia, rendering them comparable to male microglia. These sex-related morpho-functional differences may have functional consequences: it is tempting to speculate that the increased neuroprotective ability of female microglia could contribute to the 1.5–2-fold reduced risk of developing idiopathic PD observed in female individuals, a sex bias which appears to be reduced in GBA1-PD patients (Figure 7) [64,65,66,67,68]. Indeed, the majority of studies report higher female prevalence in GBA-PD or do not observe sex-related differences [65,66,67,68], suggesting that the protective effect associated with female sex is indeed blunted by GBA mutations [66]. However, a firm explanation of this difference with idiopathic PD has not been reported. Our data suggest that the differential effects of these mutations on the microglial phenotype might contribute to the differences observed between idiopathic and GBA-PD in terms of loss of male predominance.

## 5. Conclusions

We report a novel methodological approach toward the identification of dysfunctions of microglia in models of neurological diseases. The morpho-functional method was revealed to be sufficiently sensitive to recognize phenotypic differences in unstimulated microglia derived from the brain of male or female animals. Moreover, the technique demonstrated the existence of discrete subpopulations of microglia, each characterized by specific morphological descriptors, indicative of different specific phenotypes and a differential response to specific stimuli. This novel perspective provides insight into the microglial heterogeneous behaviors that might underlie pathological stimuli in different CNS regions or in the function of sex and age [69]. Indeed, the identified morpho-functional parameters allowed us to describe the morphological changes induced, not only by a well-known pro-inflammatory agent (LPS), but also by the CBE model of reduced GCase activity and GBA1-PD. Our data, for the first time, demonstrates that GCase inhibition triggers a specific microglial morpho-functional phenotype associated with a reduced ability of microglia to perform neuroprotective functions, with more dramatic consequences for microglia isolated from female animals. This finding might contribute to the understanding of the sex-related differences clinically observed in idiopathic PD.

## Figures and Tables

**Table 1:** For each parameter, using the median as a cut-off, we generated two groups named as reported in the table. Therefore, a microglial cell is included in one or the other group according to the value of the descriptor if it is bigger or lower with respect to the median value.

	Groups Generated	
Parameter	Under the Median	Over the Median
Median area	Big	Small
CV% area	Inactive	Contractile
Median solidity	Complex	Simple
CV% solidity	Steady	Variable
Median motility	Static	Motile
Median rotation	Stationary	Rotant

## Data Availability

The data that support the findings of this study are available at DOI 10.5281/zenodo.7360295. The R script is available at DOI 10.5281/zenodo.7541954. The following protocols are available at protocols.io: Luciferase activity assay (DOI: dx.doi.org/10.17504/protocols.io.j8nlkw8bxl5r/v1); cell treatments (DOI: dx.doi.org/10.17504/protocols.io.ewov1o98plr2/v1); and fluorescent image acquisition and processing (DOI: dx.doi.org/10.17504/protocols.io.ewov1o98plr2/v1). Other information is available from the senior author (paolo.ciana@unimi.it) upon reasonable request.

## Supplementary Materials

The following supporting information can be downloaded at: https://www.mdpi.com/article/10.3390/cells12030343/s1, Table S1: cluster generated for each couple of parameters, Figure S1: primary cortical cells layer composition to support microglial function, Video S1: method description, Video S2: representative time lapses of male GFP marked microglia after vehicle or LPS treatment, Video S3: representative video of the population of microglia that increase after CBE treatment.
