# Supplementary material for: Sex-Specific Microglial Responses to Glucocerebrosidase Inhibition: Relevance to GBA1-Linked Parkinson’s Disease
Source: Cells. 2023 Jan 17;12(3):343. doi: 10.3390/cells12030343 (PMC9913749; doi:10.3390/cells12030343)
Supplement: Supplementary file 1 [file cells-12-00343-s001.zip › Brunialti et al Supplementary Material.pdf]

Title: Sex-Specific Microglial Responses to Glucocerebrosidase Inhibition: Relevance to GBA1-Linked Parkinson's Disease

Authors

Electra Brunialti [1], Alessandro Villa [1], Marco Toffoli [2,3], Sara Lucas Del Pozo [2,3], Nicoletta Rizzi [4], Clara Meda [1], Adriana Maggi [5], Anthony H. V. Schapira [2,3] and Paolo Ciana [1,*].

This PDF file includes:

Supplementary table S1

Supplementary figure S1

Supplementary table S1: cluster generated for each couple of parameters.

COUPLE OF PARAMETERS		CLUSTERS GENERATED
Area-Motility	Area↓ Motility↓	Small & Static
	Area↓ Motility ↑	Small & Motile
	Area↑ Motility ↓	Big & Static
	Area↑ Motility ↑	Big & Motile
CV% Solidity-Motility	CV% Solidity↓ Motility↓	Steady & Static
	CV% Solidity↓ Motility↑	Steady & Motile
	CV% Solidity↑ Motility↓	Variable & Static
	CV% Solidity↑ Motility↑	Variable & Motile
CV Area - Solidity	CV Area↓ - Solidity↓	Inactive & Complex
	CV Area ↓- Solidity↑	Inactive & Simple
	CV Area↑ - Solidity↓	Contractile & Complex
	CV Area↑ - Solidity↑	Contractile & Simple
Solidity - Area	Solidity↓ Area↓	Complex & Small
	Solidity ↓ Area↑	Complex & Big
	Solidity ↑ Area↓	Simple & Small
	Solidity↑ Area↑	Simple & Big
Solidity - Motility	Solidity↓ Motility↓	Complex & Static
	Solidity↓ Motility↑	Complex & Motile
	Solidity↑ Motility↓	Simple & Static
	Solidity ↑ Motility↑	Simple & Motile
Rotation - Motility	Rotation↓ Motility↑	Stationary & Static
	Rotation↓ Motility↓	Stationary & Motile
	Rotation ↑ Motility↑	Rotant & Static
	Rotation ↑ Motility↓	Rotant & Motile

↓ values under the median; ↑ values over the median

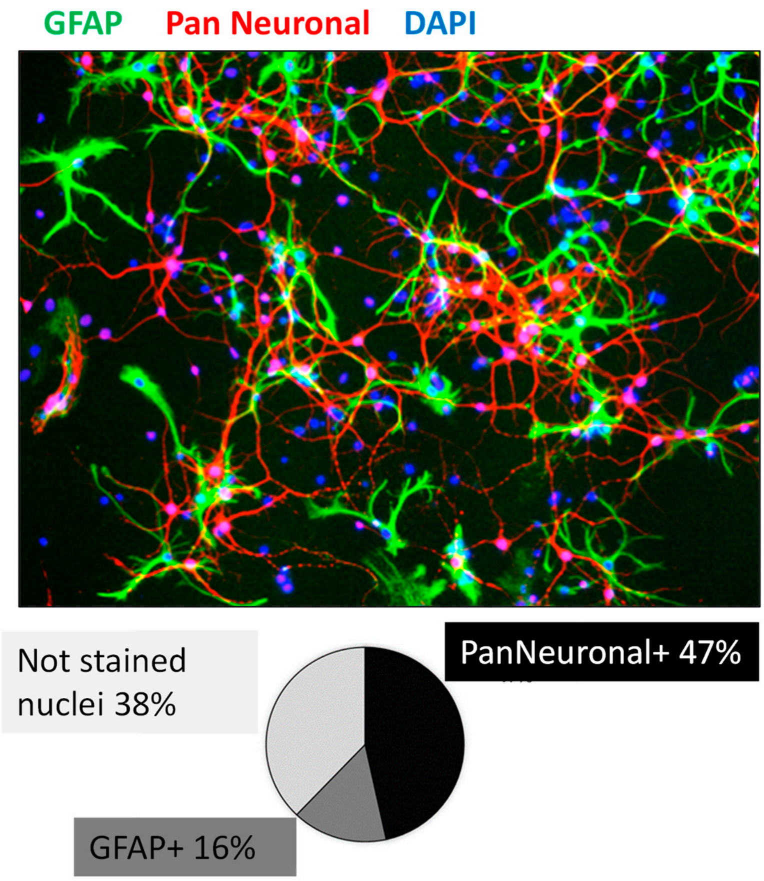

Supplementary figure S1: primary cortical cells layer composition to supports microglial function. Top) representative fluorescent image of the cortical neuronal cultures. green= GFAP marker, red=pan-neuronal marker, blue=DAPI. Bottom) the percentage of cell composition is reported with respect to the total nucleus counted.
