# Supplementary material for: Sex-Specific Microglial Responses to Glucocerebrosidase Inhibition: Relevance to GBA1-Linked Parkinson’s Disease
Source: Cells. 2023 Jan 17;12(3):343. doi: 10.3390/cells12030343 (PMC9913749; doi:10.3390/cells12030343)
Supplement: Supplementary file 1 [file cells-12-00343-s001.zip › Brunialti et al Supplementary Movie legends rev.pdf]

Title: Sex-Specific microglial responses to glucocerebrosidase inhibition: relevance to GBA1-Linked Parkinson disease

Authors

Electra Brunialti [1], Alessandro Villa [1], Marco Toffoli [2,3], Sara Lucas Del Pozo [2,3], Nicoletta Rizzi [4], Clara Meda [1], Adriana Maggi [5], Anthony H. V. Schapira [2,3] and Paolo Ciana [1,*]

This PDF file includes:

Legends for Movie 1-3

Video S1: Method description. GFP-marked microglia purified from the brain of adult mice was grown on a neuronal layer. The microglia can be easily discriminated from other cells thanks to their intrinsic fluorescence. The fluorescent images were segmented and analyzed with Fiji software to obtain morphological and dynamic parameters for each microglial during the recorded timelapse.

Video S2: representative time-lapses of male GFP marked microglia after vehicle or LPS treatment. Male microglia, 24 frames, total 2 h, scale bar 10 µm.

Video S3: representative video of the population of microglia that increase after CBE treatment. Male microglia, 24 frames, total 2 h, scale bar 10 µm.
